# The Molecular Epidemiology and Phylogeny of Torque Teno Virus (TTV) in Jordan

**DOI:** 10.3390/v12020165

**Published:** 2020-01-31

**Authors:** Haneen Sarairah, Salwa Bdour, Waleed Gharaibeh

**Affiliations:** 1 Department of Biological Sciences, Faculty of Science, The University of Jordan, Amman 11942, Jordan; 2 Department of the Clinical Laboratory Sciences, Faculty of Science, The University of Jordan, Amman 11942, Jordan

**Keywords:** TTV, Torque teno virus, prevalence, transmission, phylogeny, cow, sheep, goat, horse, donkey, chicken, Jordan

## Abstract

Torque teno virus (TTV) is the most common component of the human blood virobiota. Little is known, however, about the prevalence of TTV in humans and the most common farm domesticates in Jordan, or the history and modality of TTV transmission across species lines. We therefore tested sera from 396 Jordanians and 171 farm animals for the presence of TTV DNA using nested 5′-UTR-PCR. We then performed phylogenetic, ordination and evolutionary diversity analyses on detected DNA sequences. We detected a very high prevalence of TTV in Jordanians (~96%); much higher than in farm animal domesticates (~29% pooled over species). TTV prevalence in the human participants is not associated with geography, demography or physical attributes. Phylogenetic, ordination and evolutionary diversity analyses indicated that TTV is transmitted readily between humans across the geography of the country and between various species of animal domesticates. However, the majority of animal TTV isolates seem to derive from a single human-to-animal transmission event in the past, and current human-animal transmission in either direction is relatively rare. In conclusion, animal TTV in Jordan is historically derived from human variants; however, ongoing human-animal TTV exchange is minimal and zoonotic infection seems to be of limited importance.

## 1. Introduction

Similar to virologists the world over, researchers in Jordan have customarily concerned themselves with viruses that are known, or suspected, to cause diseases to humans [1,2,3,4]. In contrast, they have largely ignored viral components of the ‘microbiota’ in healthy people. Recently, however, a number of studies have criticized this narrow approach and have advocated for a wider perspective; the ‘virobiota,’ among other components of normal human microflora, should be characterized with an eye to investigating its interactions with the host immune system for possible consequences on host health [5]. Torque teno virus (TTV) is the most common component of the blood virobiota that does not directly cause human and animal diseases [5,6]. Since its discovery in 1997, TTV has been found to be prevalent among human populations across the globe [7,8,9,10]. Furthermore, it has also been isolated from a wide array of wild and domestic animals, including non-human primates, cats, dogs, pigs, sheep, cattle, camels, horses and chickens [11,12,13,14]. TTV is a naked single stranded DNA virus, which belongs to the family *Anelloviridae*. Among the ten *Anelloviridae* genera [15,16], the genus *Alphatorquevirus* is especially widespread among human populations worldwide and has been classified into seven phylogenetic or genogroups which include at least 39 genotypes [8,17,18]. The TTV genogroups were defined using open reading frame 1 (ORF1) sequences [16]; however, it has been repeatedly demonstrated that primers specific for the 5′ untranslated region (5′-UTR) are much more efficient in TTV detection than those specific to the ORFs of the TTV genome [19,20,21,22].

A small number of studies have examined the prevalence and genetic diversity of TTV in human and nonhuman Middle Eastern populations [10,12,23,24,25]. One of these studies reported a prevalence of 90% (27/30) among Jordanian human subjects [10]. However, this study’s small sample can be hardly taken as representative of the Jordanian population, especially given that all subjects were residents of Qatar. Therefore, we conducted the present study on a relatively large sample of blood donors and animal handlers to estimate the prevalence of human TTV in different regions of Jordan. Similarly, we estimated the TTV prevalence of the most important farm animal species. Finally, we examined the phylogenetic and genetic diversity patterns in the human and animal TTV isolates with the aim of outlining molecular relatedness across species lines, constructing the population history of the virus in Jordan, and investigating the role of interspecies transmission in the epidemiology of the virus in the country.

## 2. Materials and Methods

### 2.1. Blood Samples

#### 2.1.1. Human Subjects

A total of 396 human blood samples were collected in plain tubes from two populations: (i) healthy blood donors (*n* = 362) who attended the blood banks of Princess Basma Hospital (PBH, in Irbid, the North of Jordan), Jordan University Hospital (JUH, in Amman, the Middle region of Jordan) and Al-Karak Hospital (A-KH, in Al-Karak, South of Jordan), and (ii) animal handlers (*n* = 34) working in the Faculty of Veterinary Medicine at the Jordan University for Science and Technology (FVM-JUST, in Ar-Ramtha, the North of Jordan, *n* = 11), the Agricultural Research Station of the University of Jordan (ARS-UJ, in Damiya, the Middle of Jordan, *n* = 3), and in and near the Ma‘an Veterinary Clinic, (MAC in Petra, South of Jordan, *n* = 20). All human subjects were provided with information on TTV and gave their informed consent for inclusion before they participated in the study. The study was conducted in accordance with the Declaration of Helsinki, and the protocol was approved by the Ethics Committee of JUH (Project identification code 10.2016/3113). The geographic division into South, Middle and North regions corresponds to governmental administrative divisions and vernacular usage. Human subjects were attributed to the three geographical regions of Jordan according to place of residence, even when the locality of sample collection was different. The data collected from each donor included: sex, age, blood transfusion history, area of residence, weight and height. The body mass index (BMI) of each human subject was calculated from the weight and height.

#### 2.1.2. Domestic Farm Animals

A total of 171 blood samples were collected from farm domesticates, including: (i) sheep (*n* = 11), goats (*n* = 2) and horses (*n* = 5) from FVM-JUST (North); (ii) sheep (*n* = 26), goats (*n* = 19) and cows (*n* = 21) raised in ARS-UJ (Middle); (iii) horses (*n* = 42), donkeys (*n* = 2) and goats (*n* = 12) from and near MAC (South); and (iv) chicken (*n* = 31) from the Amman City Slaughter House in Amman (Middle). The blood samples were collected from animals following standard guidelines approved by: the Graduate Studies Committees of the Department of Biology, School of Science and School of Graduate Studies at the University of Jordan; the School of Agriculture and director of ARS-UJ at the University of Jordan; and the Jordanian Ministry of Agriculture.

### 2.2. DNA Extraction

Sera were rapidly separated from whole blood samples by centrifugation at 3800 rpm/10 min, aliquoted and stored at −20 °C until use. The viral DNA was extracted and suspended in (25 μL) nuclease free water using QIAamp MinElute Virus Spin kit (Qiagen, Hilden, Germany) in accordance with the manufacturer’s instructions.

### 2.3. TTV Detection by Nested-UTR-PCR

TTV was detected in human and animal sera by nested PCR using 5′ untranslated region (5′-UTR) primers NG133, NG147, NG132 and NG134 [26]. The PCR reaction mixture (50 μL) for the first and second rounds of the nested PCR was prepared using 3 μL of viral DNA, 1.0 μM of each primer and 25 μL of Go-Taq PCR Green master mix (Promega, Madison, WI, USA). A blank containing nuclease-free water instead of DNA was included in each run of PCR. The thermal cycling conditions were set to as those reported by Cancela [27]. PCR products (110 bp) were detected in 3% agarose gel and were purified for DNA sequencing using Monarch PCR and DNA Cleanup Kit (New England Biolabs, Ipswich, MA, USA), as recommended by the manufacturer.

### 2.4. Univariate Statistical Analysis

Associations between the prevalence of TTV, on the one hand, and geography, sex and blood transfusion history, on the other were tested using the chi-square (χ2) test, calculated from the frequency data. For the continuous variables: age, weight, height and BMI, the mean differences between the positive and negative groups were tested using exact and permuted Student’s t-tests [28]. All statistical tests were carried out in PAST3 at an α level of 0.05, adjusted for multiple comparisons using the Bonferroni and Šidák corrections [28]. PAST3 was also used to calculate 95% confidence intervals for all sample frequency estimates.

### 2.5. DNA Sequencing and Analysis

A total of 154 human isolate PCR products (124 from blood donors and 30 from animal handlers) representative of the geographical distribution of the human subjects were selected for sequencing using the NG132 and NG134 primers and the Genetic Analyzer 3500 series (Applied Biosystem, Beverly, MA, USA). All fifty animal isolate PCR products were similarly sequenced. The 5′-UTR sequences were used in subsequent analyses due to difficulty in sequencing ORF1 for our human TTV isolates and the unavailability of ORF1 sequences for donkeys, goats, sheep, cows and chicken in GenBank.

The DNA sequences of each isolate were checked against the GenBank nucleotide data base and were confirmed to be TTV using the Megablast program optimized for highly similar sequences in the Basic Local Alignment Search Tool (BLAST) of the National Center for Biotechnology Information (NCBI, National Institutes of Health, USA). The electropherogram results of forward and backward sequences were edited and assembled into contigs using the Molecular Evolutionary Genetics Analysis program (MEGA) version 7.0 [29].

The matrix sequence of identity percentage was calculated for human and animal PCR products using the BioEdit sequence alignment editor software version 7.1.9 [30]. The sequences of the PCR products were aligned against the UTR sequences of TTV genogroups 1–5 and 7 retrieved from GenBank using Multiple Sequence Alignment with High Accuracy and High Throughput (MUSLE) in MEGA7 [29]. Genogroup 6 was not included in the analysis because the UTR sequence of this group was not available in GenBank. The following are the accession numbers for the “reference” sequences of TTV genogroups 1–5 used in this study: AB017610, AB017613, AB025946, AB028669, AB054647, AB054648, AB064595, AB064596, AB064601, AB064604, AB064605, AB064606, AB064607, AF116842, AF122913, AF122914, AF122915, AF122916, AF122918, AF122921, AF247137, AF247138, AF261761, AF435014, AJ620212, AJ620225, AJ620226, AJ620227, AM711976, AY823988, DQ186996, DQ186999, FR751472, FR751476, FR751488, FR751500, GU797360, KJ082064, AB273627, AB273631, AB273632, FR848325, FR751493, FR848324. KF477315 is the accession number for the outgroup sequence.

### 2.6. Phylogenetic, Ordination and Diversity Analyses

Principal coordinate analysis (PCoA) was implemented for the visualization of the genetic relationships between human blood donor, animal handler and animal TTV isolates using the software PAST3 [28]. The principal coordinates were computed on the identity matrix generated by BioEdit [30]. Principal coordinates analyses on distance matrices generated under the Kimura 2-parameter [31] and composite likelihood models [32] were also computed and gave similar results. The phylogenetic relationships between the human and animal TTV samples were reconstructed in the form of a Neighbor-Joining tree [33], computed on the pair-wise composite likelihood distances [32], and rooted using the pigeon Torque teno virus (PTTV) sequence [34] as an outgroup and implemented in the MEGA7 package [29]. Using the STTV CH65-1 simian TTV sequence as an outgroup for rooting the tree yielded the same general patterns in terms of the relationships across geographic and species lines. Gaps and missing data were treated with pairwise deletion. Genogroups of human and animal TTVs were provisionally estimated, if possible, from the tree based on the clustering with the reference TTV isolates from GenBank.

Evolutionary analyses of genetic distance and diversity were also computed in MEGA7 [29]. Mean evolutionary distances within and between pooled human, and animal isolate samples (regardless of the species) were estimated under the Kimura2-parameter model [31] using the number of transitional and transversional base substitutions per site after eliminating ambiguous positions from each nucleotide sequence pair. Standard error estimates (SE) were obtained by using a 1000 replicate bootstrap procedure. Similarly, the estimates of mean diversity within group samples (human isolates and animal isolates), between group samples (human/animal isolates), and for the entire sample of isolates were computed in MEGA7 [29] using the number of transitional and transversional base substitutions per site under the Kimura 2-parameter model [31]. The same model was also used to estimate between-group (human/animal isolates) evolutionary differentiation coefficients [35], in MEGA7 [29]. As before, ambiguous positions were removed for each sequence pair and standard errors (SE) of distance, diversity and differentiation estimates were computed using a 1000 replicate bootstrap procedure. The same mean evolutionary distance and diversity and differentiation estimates were computed for the subset of human isolates in order to make blood donor vs. animal handler, and North vs. Middle vs. South comparisons.

## 3. Results

### 3.1. High TTV Prevalence in Jordanians

The overall prevalence of TTV in Jordanians is 95.5% (378/396) (Table 1). The difference in TTV prevalence between blood donors (96.1%) and animal handlers (88.2%; Table 1) is statistically significant when calculating the probability from the χ2 distribution (χ2 test statistic = 4.4676, df = 1, *p* = 0.034543), but not when using the more robust permutation test with 99999 permutations (*p* = 0.05881). The difference is not significant, no matter how the *p* is calculated, when adjusting for multiple comparisons (correcting for nine study-wide comparisons yields an adjusted α_0.05_ = 0.0056 or 0.0057 using the Bonferroni and Šidák corrections, respectively. Therefore, formally these two human subgroups could be pooled and treated as one. The blood donor subgroup (*n* = 362) is probably a better representative of the Jordanian population as a whole than the pooled data, given that animal handlers are overrepresented in the pooled sample compared to their numbers in the population. Regardless, the estimated frequencies are very similar (96.1% vs. 95.5%) and limiting the subsequent analyses to this group (blood donors) gives similar results as using the pooled data.

### 3.2. Geographically Homogenous TTV Prevalence in Jordanians

A high prevalence of human TTV was detected throughout Jordan (Table 2). The prevalence in the densely populated northern and middle regions is identical to the nearest percent point, and the prevalence in the sparsely populated southern region is only few percent points less. The difference in TTV prevalence among the three regions of Jordan is not statistically significant (χ2 = 3.9404; *p* = 0.13943) nor are the differences between pairs of regions, even without correcting for multiple comparisons (North vs. Middle: χ2 = 0.0022, *p* = 0.96017; Middle vs. South: χ2 = 3.3749, *p* = 0.066197; North vs. South: χ2 = 2.0746, *p* = 0.14977). The sample sizes are not proportional to the population sizes of the three regions; thus, the Middle region sample is probably a better representative of TTV prevalence in Jordan overall (given that the majority of Jordanians live in the Middle region) than pooling the three regions together. Regardless, the difference in prevalence between the pooled sample and the Middle region alone is inconsequential (95.5% vs. 96.5%, respectively; Table 2).

### 3.3. Demographically Homogenous TTV Prevalence in Jordanians

A total of 375 male and 21 females aged 18–58 years were enrolled in the study (Table 3). The overall prevalence of TTV in males (95.47%) is almost identical to that of females (95.24%); the minute difference is not statistically significant (χ2 = 0.0024; *p* = 0.96097). The difference in TTV prevalence between blood donors who had not received blood transfusion (95.73%) and those who had received blood transfusion (90.48%) is not statistically significant (χ2 = 1.2667; *p* = 0.26038). Furthermore, the relationship between testing positive to TTV and the subject characteristics including age, BMI, weight and height is not statistically significant in all cases (age: *t* = −0.12755, *p* = 0.89857; BMI: *t* = 0.54464, *p* = 0.58631; weight: *t* = 0.90123, *p* = 0.36802; height: *t* = 0.91514, *p* = 0.36068).

### 3.4. Relatively Low Prevalence of TTV in Animals

The overall prevalence of TTV in animals is estimated at 29.24% with a 95% confidence interval of 22.94%–36.45% (Table 4). The highest prevalence of TTV is in equids (the 63.83% prevalence in horses is more indicative than the 100% prevalence in donkeys, for which *n* = 2) and the lowest is in cows (4.76%). The difference in TTV prevalence among animals is statistically significant (χ2 = 54.676; *p* = 1.5217 × 10^−10^; Bonferroni-adjusted α_0.05_ = 0.008).

### 3.5. Ordination, Phylogenetic and Diversity Analyses 

**Figure 1:**
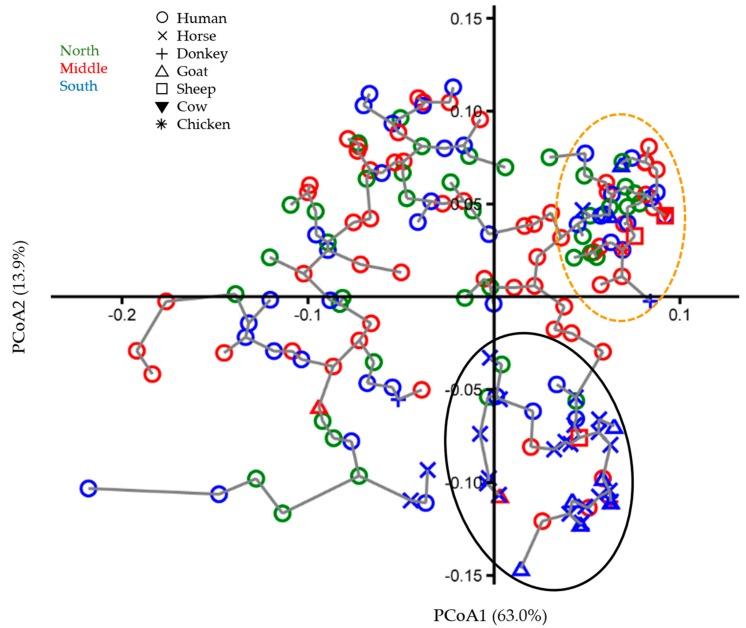
Principle coordinates analysis plot (principal coordinate 1 vs. principal coordinate 2; combined percent variance explained = 77%), with minimum spanning tree, computed on the identity matrix for human and animal TTV isolates’ 5′-UTR sequences. The geographical locations of isolates are in different colors. Isolates from different host species are represented by different symbols.

**Figure 2:**
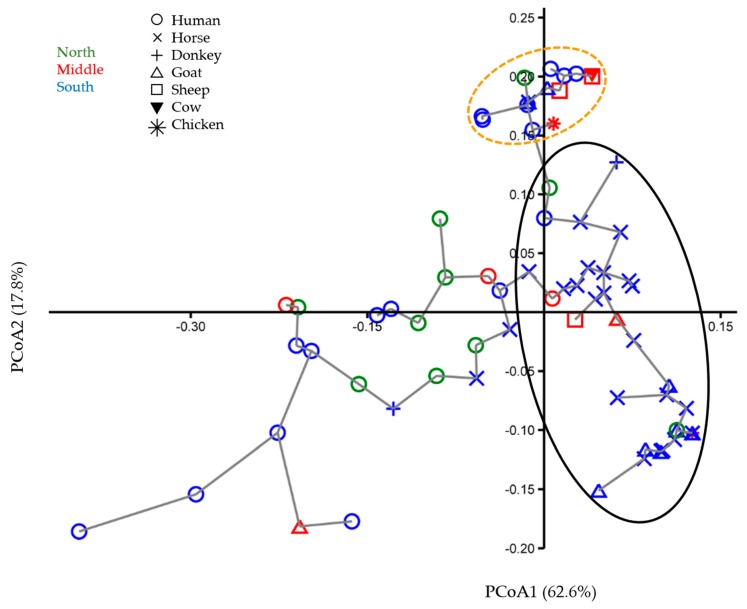
Principle coordinates analysis plot (principal coordinate 1 vs. principal coordinate 2; combined percent variance explained = 80%), with minimum spanning tree, computed on the identity matrix for animal handler and animal TTV isolates’ 5′-UTR sequences. The geographical locations of isolates are in different colors. Isolates from different host species are represented by different symbols.

The results of multivariate ordination, phylogenetic reconstruction and evolutionary distance and diversity analyses are largely congruent, all being reflections of the same population history and transmission patterns. The following patterns can be observed in the ordination analysis: first, the human TTV isolate variation dwarfs that of the animal isolates, which are largely concentrated in one corner of the plot (Figure 1, oval demarked by a solid black line). This pattern is not merely the consequence of the larger number of human isolates (see diversity analyses below). Out of the fifty sequenced animal TTV isolates, thirty-nine (78%; including one chicken, nine goats, one sheep, one donkey, and twenty-seven horses) cluster distinctly (Figure 1, black solid line oval) from the majority of human TTV isolates (142 out of 154 sequenced). In other words, only 12 human TTV isolates (7.8% of 154) cluster with the majority (78%) of animal isolates. At the same time, the animal isolates are closely related to the human isolates and can be viewed as being positioned at the margin of the envelope of human isolate diversity. Second, of these 12 human TTV isolates found in the tight animal isolate cluster, eight are from blood donors (out of 134, 6.5%) and four (out of 30, 13%) are from animal handlers (Figure 1 and Figure 2, black solid line ovals). Compared to blood donors, animal handlers have a lower TTV prevalence in general (88.2% vs. 96.1%). However, they have a significantly higher frequency of the TTV isolates most commonly found in animals (χ2 = 24.059; *p* = 9.3443 × 10^−7)^. The principal coordinates plot of animal handler and animal isolates shows that the handler isolates’ diversity is larger than that of animal isolates (Figure 2), even though they are fewer in number (*n* = 30 vs. *n* = 50). Third, no geographic clustering of human TTV isolates is evident in the principal coordinates plot (Figure 1). Fourth, although the animal isolates as a group cluster largely separate from human isolates, the distribution of isolates within this cluster does not reflect the phylogenetic relationships of the host species. For example, goats and sheep belong to subfamily Caprinae in the order Cetartiodactyla and horses and donkeys belong to same genus, *Equus*, in the order Perissodactyla; however, TTV isolates from goats and horses cluster tightly together instead of clustering with isolates from their close relatives (Figure 1 and Figure 2). Fifth, within the envelope of human isolate diversity, there is a particularly dense tight cluster of a large number of human isolates along with a few animal isolates (Figure 1 and Figure 2, orange dashed line ovals).

**Figure 3:**
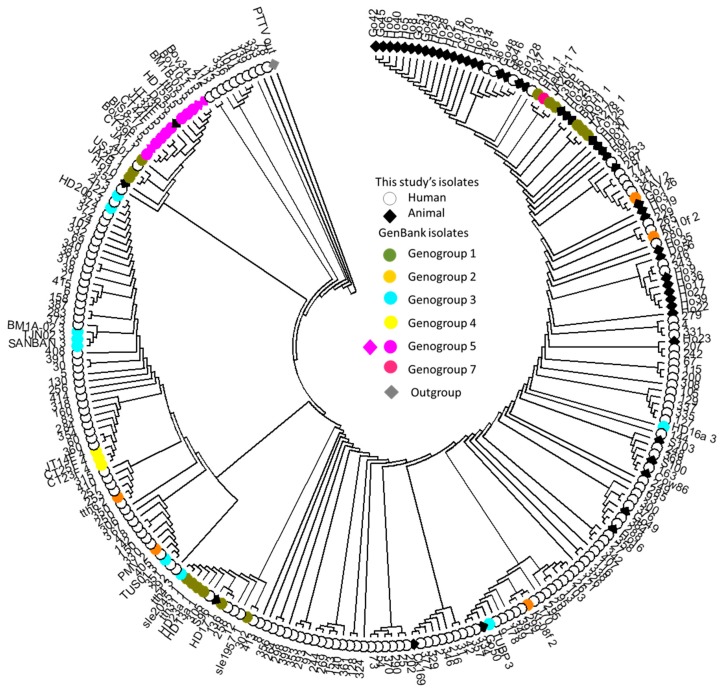
Phylogenetic tree constructed by the neighbor-joining method based on the 5′-UTR for human and animal TTV isolates. The evolutionary history was inferred using the Neighbor-Joining method and rooted using the pigeon Torque teno virus (PTTV) sequence as an outgroup. The evolutionary distances were computed using the Maximum Composite Likelihood method and are in the units of the number of base substitutions per site. The analysis involved 249 nucleotide sequences (50 animals, 154 human and 45 from GenBank representing 6 common genogroups). All ambiguous positions were removed for each sequence pair, leaving a total of 125 positions in the final dataset. Abbreviations: Ho: horse; S: sheep; Go: goat; Ck: chicken; Do: donkey. Human isolates are represented by circles and animal isolates by diamonds.

Similar to the findings in the ordination analysis, the majority of animal TTV isolates (36/50) cluster closely together in a tight clade in the phylogenetic tree (Figure 3), which is almost the same in its membership as the cluster delineated by the black solid line ovals of the principal coordinates plot in Figure 1 and Figure 2. Moreover, this animal isolate cluster nests within a larger clade of lineages comprising mostly human isolates. To extend the analogy further, the animal TTV isolates do not segregate into species-specific clades, nor do they reflect the phylogeny of the hosts in any way in the phylogenetic tree. Interestingly, the five most basal lineages in the tree are exclusively comprised of ten Jordanian isolates. In terms of identifying our isolates with established genogroups, the human TTV isolates clustered with the GenBank reference isolates belonging to genogroups 1, 2, 3, 4, 5 and 7, while animal TTV isolates clustered with the reference isolates of genogroups 1, 2, 3, 5 and 7 (Figure 3).

**Figure 4:**
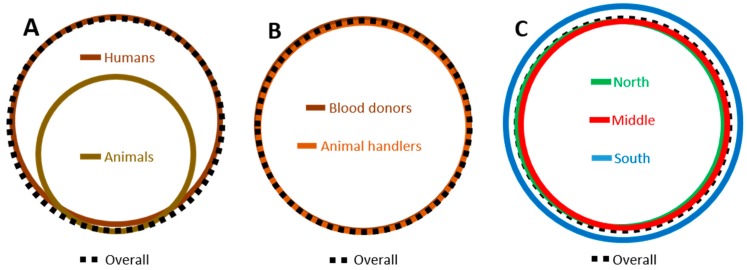
A schematic representation of the 5′-UTR sequence evolutionary distance and diversity analyses. Panels (**A**–**C**) represent Table 5, Table 6, Table 7, respectively. The area of each circle is proportional to the mean pairwise evolutionary distance.

The evolutionary distance and diversity analyses help us quantify and confirm the statistical significance of the ordination and phylogenetic analyses findings (Table 5, Table 6, Table 7; Figure 4). The mean evolutionary pair-wise distance within the human TTV isolates (0.069; Table 5) is almost twice that for animal isolates (0.038; Table 5). The apportionment of total sample diversity (0.072) between human and animal isolates shows that most of variation is within group (0.054; Table 5); however, there is a significant mean inter-subsample diversity (0.019 ± 0.005) and coefficient of differentiation (0.258 ± 0.028). Figure 4A is a schematic representation of Table 5, in that it shows a circle representing human isolate diversity with nearly twice the area of a circle which represents the diversity of the combined animal isolates. The smaller animal isolate circle is largely contained within the human isolate circle and a considerable off-center displacement represents the differentiation between the two groups.

Conversely, blood donor and animal handler isolates are identical in their mean evolutionary distance (0.071; Table 6). Apportioning the human isolate diversity into blood donors and animal handlers shows that none of the human isolate diversity can be attributed to differences between those two groups (zero mean inter-subsample diversity and non-significantly negative coefficient of differentiation)—the within group differences accounting for all of the sample diversity (Table 6). These results are illustrated in Figure 4B, in which the circles representing blood donor, animal handler and total human isolate diversities are almost identical in size and nearly perfectly concentric.

Similarly, the mean evolutionary distance for blood donor isolates from the North of the country is identical (0.068) to that for donor isolates from the Middle, and the mean evolutionary distance for North-Middle pairs is identical to those within-region for these two regions (Table 7). The mean evolutionary distance for the isolates from the less densely populated South is slightly higher, but not significantly so (0.088 ± 0.019; Table 7) and the mean pairwise evolutionary distances between the South isolates and those of the two other regions is similarly slightly, but not significantly, higher than the mean North-Middle evolutionary distance (Table 7). Apportioning the blood donor isolate diversity geographically shows that none of it can be attributed to the inter-region component (non-significantly negative mean inter-subsample diversity and coefficient of differentiation). Figure 4C illustrates this result with circles representing the North, Middle, South and total isolate diversities that are almost equal in size and nearly perfectly concentric.

## 4. Discussion

The current study is the first of its kind on the prevalence of TTV in either humans or animals in Jordan. It is also the first study to investigate the geographic patterning of TTV prevalence and genotypes in humans within a country in the Middle East and North Africa. In addition to gauging prevalence, this study investigates the molecular relatedness between Jordanian TTV isolates and those from their most important farm domesticates with the aim of gaining insights into the within-species and inter-species modes of transmission.

The high prevalence of TTV among Jordanians in all parts of Jordan (91.36%–96.58%; Table 2) is slightly higher than that reported for the small sample of Jordanians residing in Qatar (90%; *n* = 30) [10]. Within the relatively narrow confines of Jordan (the farthest samples are less than 450 km apart), no significant differences in prevalence among regions were detected. This is to be contrasted with a number of studies of much larger countries which reported geographical variation in TTV prevalence (3%–72% in India [36,37]; 2%–34% in Iran [9,23] and 6%–85% in Brazil [38,39,40]). The fact that these wide ranges were reported by different studies per country, leaves open the possibility that the observed within-country difference in prevalence might be at least partly due to variation in methodology (sampling scheme, sets of primers used, year of study, etc.) and not just the larger geographic scale. The sampling scheme of animal isolates did not allow for the investigation of differences in prevalence among regions within an animal species.

We did not detect a significant relationship between TTV prevalence and the subjects’ characteristics, including sex, blood transfusion history, age, height and BMI (Table 3). The prevalence is almost identical between males and females (95.5 and 95.2%, respectively); thus, the lack of statistical significance cannot be attributed to lack of statistical power due to relatively small sample of females (*n* = 20). Similarly, the difference in prevalence between individuals who had a history of receiving blood transfusions and those who did not (90.5% and 95.7%, respectively) is also small in magnitude and in the opposite direction to that expected if blood transfusions were responsible for introducing TTV to the subjects. Similar to our findings here, a number of studies failed to find a significant relationship between TTV prevalence and subject characteristics in other countries, including Egypt [41], Russia [42], Brazil [40], Iran [23] and Qatar [10]. Conversely, a number of studies reported a significant association between TTV prevalence and subject attributes in other countries, including: gender [sic] in the UAE [43]; blood transfusion history in Iran [23]; age in the UK [44], China [19], Brazil [40] and Qatar [10]; and obesity in Romanian women [45].

The overall TTV prevalence in farm animals sampled in this study was considerably lower (29.24%, Table 4) than that for Jordanians. The highest prevalence was recorded in equids, with 63.83% of horses testing positive (TTV was also detected in the two donkeys tested), followed by goats (36.36%). The prevalence of TTV in the rest of the animal species was in single digit percent points. It should be noted here that this is the first report on the presence of TTV in donkeys and goats anywhere in the world, which makes these isolates prime candidates for sequencing and genome characterization. The TTV prevalence in this study’s horse sample was higher than the 12.3% reported for Californian diseased horses [14]. However, our results for all the other animal species show a prevalence that is lower than that reported in the literature for other countries: viz. for sheep (8.11% vs. 30% in the USA [46]); for cows (4.8% vs. 25% in the USA [46] and 42% in Canada [13]); and for chicken (6.5% vs. 19% in USA [46] and 28% in Iran [24]). The number of TTV studies in domesticated animals remains too small to draw broad conclusions—especially considering that a number of other studies failed to detect TTV in domesticates at all, including UK cows, sheep, goats and chicken [47] and Brazil chicken [48].

It is possible that low observed prevalence in domestic animals compared to humans in this study is due to our use of PCR primers that have been reported to be effective for detection of TTV in humans and that the design and employment of animal host species-specific PCR primers could reveal a much heavier presence of unknown TTV variants in animals that could not be detected by the primers used in this study and the literature at large. There are indeed reports of animal TTV variants very different from the ones common in humans; for example, TTV Equ1 isolated from horses was found to be distantly related to the genus *Alphatorquevirus* TTV isolates and was proposed as a prototype of a new genus, *Mutorquevirus* which occupies a basal position within the family *Anelloviridae* [14]. The subject of this study, however, is the *Alphatorquevirus* TTV isolates found commonly in humans and their modes of transmission within and between humans and other putative host animals, with a special focus on possible zoonotic transmission.

In the current study’s sample, not only is Jordan’s overall prevalence of TTV in animals appreciably lower than in humans, but also the genetic variance among animal isolates is much more limited than that of the human isolates. This is illustrated in Figure 1, in which the animal TTV isolates occupy a small corner of the principal coordinate space occupied by the human isolates. Figure 2 further demonstrates the great diversity among human isolates as represented by the animal handler isolates alone; these isolates occupy a larger ordination space than that of the more numerous animal isolates. The same pattern is observed again in the phylogenetic tree (Figure 3), in which the vast majority of TTV animal isolates cluster in one clade that is closely related to the clades containing the vast majority of human TTV isolates. This pattern is also reflected in the narrow range of genetic identity among animal TTV isolates (74.7%–100%) despite the fact that they are taken from host species belonging to different species, families, orders and even classes, compared to that in isolates taken from one human population (60.8%–100%). This means that the maximum observed genetic divergence is larger among human TTV isolates (60.8%) than among their animal counterparts across species lines (74.7%). Furthermore, the range of genetic identity for human–animal pairs (67.7%–100%) is narrower than that for human–human pairs, which indicates that the genetic variation of the animal TTV isolates is largely contained within that of the human isolates. The evolutionary distance and diversity analyses of the total sample divided into human isolate vs. animal isolate subsamples quantify these patterns and confirm their statistical significance (Table 5; Figure 4A). The observation that human isolates are of much greater diversity than animal isolates is substantiated by the fact that the mean within-human isolate evolutionary distance (0.072) is almost twice that for animal isolates (0.038). The evolutionary diversity analysis further confirms that there is great deal of overlap in the diversity of the two groups, as demonstrated by the relatively large mean within group diversity (0.054), but that there is a significant inter-group diversity component (mean inter-subsample diversity = 0.019 ± 0.005; coefficient of differentiation = 0.258 ± 0.028). Together, all of these observations strongly indicate that the majority of the animal domesticate TTV isolates in Jordan originated from a single human-to-animal transmission event.

Compared to blood donors, animal handlers have a slightly lower prevalence of TTV in general (88.2% vs. 96.1%), which might reflect their rural and sparsely populated surroundings. However, the latter were found to harbor twice the frequency of the TTV isolates that are most common among animals compared to blood donors (13 vs. 6.5%; χ2 = 24.059; *p* = 9.3443 × 10^−7^). This result suggests some “back” transmission from animals to humans that is statistically significant but limited in scope. The diversity analysis of the human TTV sample divided into blood donor vs. animal handler groups shows a negligible between-group component of diversity (Table 6 and Figure 4B). This further suggests that the animal-to-human transmission, albeit statistically detectable, occurs too rarely to register differentiation between animal handlers and the general population (represented by blood donors). Collectively, these observations indicate that the vast majority of TTV infections detected in Jordanian animal handlers were contracted from fellow humans and not from contact with the animal domesticate species sampled in this study. This interpretation is consistent with the lower TTV infection rate in animal handlers, who on average are expected to have lower rates of human interactions than the majority of the population concentrated in the urban conurbations of the country.

The relative ease of TTV transmission between human subjects is reflected in the lack of genetic differentiation between geographic regions in Jordan (Table 7 and Figure 4C). Even the tight cluster of mostly human isolates which represents a recent TTV “outbreak” (demarked by orange dashed line ovals in Figure 1 and Figure 2) is geographically mixed in its TTV isolate composition. This is not surprising, considering the narrow geography of this relatively small country, its high urbanization rate (90.75% in 2017) [49] and the fact that a considerable proportion of regional population commutes daily to the urban centers in the Middle and North of the country for work and most of the rest frequently travel to the cities for administrative, business and other reasons. The conclusion that human-to-human TTV transmission occurs readily, relatively speaking, through everyday interactions is also consistent with the result that history of blood transfusion does not contribute to the high infection rate among Jordanians. As far as the animal domesticates is concerned, the conclusion that most of the animal TTV isolates seem to derive from a single human-to-animal transmission event with little differentiation by animal host means that it is much more likely for animal domesticates to contract TTV from other domesticates (even those belonging to distantly related species) than to contract it from humans. This is consistent with reports from other countries in which TTV isolates from farm animals including chicken, cow, sheep and pig did not group into species-specific clusters [46].

Collectively, the observations above point to a historical scenario for Jordan in which TTV spread into humans first and then from them to their farm domesticates. There seems to have been some other instances of human-to-animal transmission events (as evidenced by the few animal TTV isolates scattered out and away from the cluster marked by black solid line ovals, Figure 1 and Figure 2) as well as instances of animal–to–human TTV transmissions (as evidenced by the disproportional number of animal handler isolates within the cluster marked by orange dashed line ovals, Figure 1 and Figure 2); however, the human–animal domesticate transmission rate in both directions is considerably lower than the human–human transmission rate and animal–animal rate (even across species). This pattern strongly weakens the consideration of TTV as a zoonotic virus, at least with regards to the animal species considered in this study. Furthermore, the likelihood of an unstudied animal source contributing significantly to the high TTV infection rate in Jordan is small, given the fact that the animals studied here are the most important farm domesticates in the country, the limited scope of the practice of pet keeping in the country and how the predominantly urban population of Jordan rarely comes into contact with any other kind of animal. In contrast to our findings here, sixteen camel and thirteen human TTV isolates from UAE did not cluster into species-specific clades in a 5′-UTR neighbor-joining phylogenetic tree, but rather intermingled in mixed clusters, suggesting the possibility of frequent cross-species transmission [12]. Assuming that this result is biologically meaningful, and not just a reflection of a weak phylogenetic signal, it indicates a much-elevated camel-human TTV transmission rate compared to that between humans and the domesticates of this study. Furthermore, a number of studies have reported evidence for zoonotic or reverse zoonotic TTV transmission between humans and other primates [11,48]. This is not surprising, considering the close evolutionary relationship in that case; however, this route of transmission is not relevant to the TTV epidemiology in Jordan, since there are no extant wild primates native to Jordan and primate pets are extremely rare in the country.

Genogroup 3 is modal in our identifiable human isolates, as it is worldwide [10,50]. Similarly, the majority of TTV isolates from Jordanians residing in Qatar, were identified with genogroup 3 (70%), with the remainder identified with genogroup 5 [10]. However, it was not always easy to match the sequences of 5′-UTR to a specific TTV genogroup given that these genogroups were defined using ORF sequences. The 5′-UTR phylogenetic trees are not congruent with ORF phylogenetic trees and, as such, isolates known to belong to particular genogroups do not come out monophyletic when clustered using their UTR sequences. The lack of agreement in the phylogenetic signals of ORF and UTR sequences is not particular to our Jordanian TTV sample as it was also reported for USA [51], UK [11], UAE [12] and Qatar [10] and is possibly due to frequent recombinations in the UTR [52]. While identifying our isolates’ genotypes would have been gratifying, it is not necessary for the purposes of this study. The analysis of the diversity patterns of the 5′-UTR sequences showed unequivocal patterns that are highly significant statistically. Interpreting these patterns as anything other than a reflection of the TTV population history invokes scenarios that are interesting in their own right, but which are too elaborate and improbable, given the data.

## 5. Conclusions

TTV prevalence in Jordanians is very high, comparable to that reported for many other human populations. For the studied farm animal domesticates, the prevalence of TTV variants targeted by the primers used in this study is much lower than in Jordanians. Multivariate ordination, phylogenetic, and evolutionary distance and diversity analyses suggest a most parsimonious reconstruction for the history of *Alphatorquevirus* TTV and modality of transmission in Jordan in which the virus had a relatively long residence in the local human population before passing on to their domesticates. TTV is transmitted readily between humans across the geography of the whole country and between various species of animal domesticates. However, the rate of human-animal transmission in both directions is relatively low. In other words, most human TTV infections are contracted from fellow humans and most animal infections are contracted from animals, but not necessarily from the same species. The TTV 5′-UTR phylogenetic signal was found to be too weak to allow for a complete and unequivocal identification of our isolates with the genogroups established using the ORF1 phylogeny. This recommends the sequencing the complete genome of selected human and animal TTV isolates from our sample as an important next step which might assist us in: (1) drawing more definite conclusions regarding the phylogenetic relations of these isolates and their genogroup affiliations; (2) conducting a thorough investigation of the TTV phylodynamics and modes of transmission across geography and host species lines in Jordan and globally; (3) designing animal specific TTV PCR primers, which might allow us to detect and collect a larger number of TTV isolates from a variety of animal domesticate and wild species in Jordan. This would give a more sensitive gauging of animal TTV loads and possible hidden dimensions of inter-species transmission. This is especially important for the TTV genomes of goat, chicken, cow, sheep and donkey, which are yet to be characterized in any study.

## Figures and Tables

**Table 1:** The prevalence of TTV in Jordanian blood donors and animal handlers.

Sample	No. Tested	Positive		Negative		95% CI * for % Positive	
		No.	%	No.	%	LCL *	UCL *
Blood donors	362	348	96.1	14	3.9	93.61	97.68
Animal handlers	34	30	88.2	4	11.8	73.38	95.33
Total	396	378	95.5	18	4.6	92.93	97.11

* CI: confidence interval; UCL: upper confidence limit; LCL: lower confidence limit.

**Table 2:** The prevalence of human TTV in the North, Middle and South of Jordan.

Region	No. Tested	Positive		Negative		95% CI * for % Positive	
		No.	%	No.	%	LCL *	UCL *
North	88	85	96.59	3	3.41	90.45	98.83
Middle	227	219	96.48	8	3.52	93.20	98.20
South	81	74	91.36	7	8.64	83.22	95.75
Total	396	378	95.45	18	4.55	92.93	97.11

* CI: confidence interval; UCL: upper confidence limit; LCL: lower confidence limit.

**Table 3:** Demographic characteristics of Jordanian blood donors.

Category		TTV Positive		TTV Negative		Total
		No.	%	No.	%	
Sex	Male	358	95.5	17	4.5	375
	Female	20	95.2	1	4.8	21
Blood transfusion	No	359	95.7	16	4.3	375
	Yes	19	90.5	2	9.5	21
Age (years)	18–30	194	96.0	8	4.0	202
	31–43	132	94.3	8	5.7	140
	44–58	52	96.3	2	3.7	54
Weight (Kg)	40–60	35	92.1	3	7.9	38
	61–80	165	95.9	7	4.1	172
	81–100	140	95.9	6	4.1	146
	>100	38	95	2	5	40
Height (cm)	≤ 160	28	93.3	2	6.7	30
	161–177	226	95.8	10	4.2	236
	>178	124	95.4	6	4.6	130
BMI (Kg/m^2^)	Underweight (<18.5)	3	75	1	25	4
	Healthy weight (18.5–25)	155	95.7	7	4.3	162
	Over weight (26–30)	149	94.9	8	5.1	157
	Obese (>30)	71	97.3	2	2.7	73

**Table 4:** The prevalence of TTV in domestic farm animals.

Sample	No. Tested	Positive		Negative		95% CI * for %Positive	
		No.	%	No.	%	LCL *	UCL *
Horse	47	30	63.83	17	36.17	49.54	76.03
Donkey	2	2	100	0	0	34.24	100
Goat	33	12	36.36	21	63.64	22.19	53.38
Sheep	37	3	8.11	34	91.89	2.80	21.30
Cow	21	1	4.76	20	95.24	0.85	22.67
Chicken	31	2	6.45	29	93.55	1.79	20.72
Total	171	50	29.24	121	70.76	22.94	36.45

* CI: confidence interval; UCL: upper confidence limit; LCL: lower confidence limit.

**Table 5:** Estimates and standard errors (SE) of mean evolutionary distance and diversity within and between human TTV isolates and domestic farm animal (all species) TTV isolates.

**Mean Pairwise Evolutionary Distance**		
	Human TTV Isolates ± SE	Animal TTV Isolates ± SE
Human TTV isolates ± SE	0.069 ± 0.016	0.082 ± 0.020
Animal TTV isolates ± SE	0.082 ± 0.020	0.038 ± 0.009
Overall = 0.072 ± 0.017		
**Mean evolutionary diversity for human and animal TTV isolates**		
	Diversity	SE
Mean within subsample diversity (human/animal TTV isolates)	0.054	± 0.012
Mean inter-subsample diversity (between human and animal TTV isolates)	0.019	± 0.005
Mean diversity for the entire study sample (pooled human and animal TTV isolates)	0.072	± 0.016
Coefficient of differentiation between human and animal TTV isolates	0.258	± 0.028

**Table 6:** Estimates and standard errors (SE) of mean evolutionary distance and diversity within and between blood donor TTV isolates and animal handler TTV isolates.

**Mean Pairwise Evolutionary Distance**		
	Donor TTV Isolates ± SE	Handler TTV Isolates ± SE
Donor TTV isolates ± SE	0.071 ± 0.015	0.070 ± 0.017
Handler TTV isolates ± SE	0.070 ± 0.017	0.071 ± 0.018
	Overall = 0.070 ± 0.016	
**Mean evolutionary diversity for human TTV isolates**		
	Diversity	SE
Mean within subsample diversity (blood donor/animal handler TTV isolates)	0.071	± 0.016
Mean inter-subsample diversity (between donor and handler TTV isolates)	0.000	± 0.002
Mean diversity for the entire study sample (pooled donor and handler TTV isolates)	0.070	± 0.016
Coefficient of differentiation between donor and handler TTV isolates	−0.005	± 0.029

**Table 7:** Estimates and standard errors (SE) of mean evolutionary distance and diversity within and between blood donor TTV isolates from the North, Middle and South regions of Jordan.

**Mean Pairwise Evolutionary Distance**			
	Donor TTV Isolates for North ± SE	Donor TTV Isolates for Middle ± SE	Donor TTV Isolates for South ± SE
Donor TTV isolates for North ± SE	0.068 ± 0.016	0.068 ± 0.016	0.077 ± 0.017
Donor TTV isolates for Middle ± SE	0.068 ± 0.016	0.068 ± 0.016	0.079 ± 0.018
Donor TTV isolates for South ± SE	0.077 ± 0.017	0.079 ± 0.018	0.088 ± 0.019
Overall = 0.072 ± 0.016			
**Mean evolutionary diversity for blood donor TTV isolates by region**			
		Diversity	SE
Mean within subsample diversity (North/Middle/South donor TTV isolates)		0.075	±0.017
Mean inter-subsample diversity (among North, Middle and South TTV isolates)		−0.002	±0.001
Mean diversity for the entire study sample (pooled TTV isolates from all regions)		0.072	±0.016
Coefficient of differentiation among regional TTV isolates		−0.032	±0.014
